# Coronary heart disease risk in patients with schizophrenia: a Lebanese cross-sectional study

**DOI:** 10.15256/joc.2017.7.107

**Published:** 2017-07-13

**Authors:** Chadia Haddad, Souheil Hallit, Pascale Salameh, Tarek Bou-Assi, Marouan Zoghbi

**Affiliations:** ^1^Research Department, Psychiatric Hospital of the Cross, Jal Eddib, Lebanon; ^2^Faculty of Pharmacy, Lebanese University, Beirut, Lebanon; ^3^Faculty of Pharmacy, Saint-Joseph University, Beirut, Lebanon; ^4^Faculty of Medicine and Medical Sciences, Holy Spirit University of Kaslik, Kaslik, Lebanon; ^5^Faculty of Medicine, Lebanese University, Beirut, Lebanon; ^6^Department of Laboratory Medicine, Psychiatric Hospital of the Cross, Jal Eddib, Lebanon; ^7^Department of Family Medicine, Saint-Joseph University, Beirut, Lebanon; ^*^Equal contribution.

**Keywords:** schizophrenia, cardiovascular disease, antipsychotics, lifestyle interventions, Framingham risk score

## Abstract

**Background:**

Coronary heart disease (CHD) is a leading cause of premature death in patients with schizophrenia. CHD risk in Lebanese patients with schizophrenia remains unknown.

**Objectives:**

To (i) evaluate CHD risk of patients with schizophrenia in Lebanon; and (ii) detect the modifiable and non-modifiable factors affecting this risk.

**Methods:**

Cross-sectional study of 329 patients with schizophrenia aged 20–75 years. Ten-year hard CHD risk was calculated using the Framingham risk score. A logistic regression was conducted taking the dichotomous hard CHD (<10% and ≥10%) as the dependent variable.

**Results:**

Ten-year hard CHD risk was low (<10%) in 60.8% of patients, intermediate (10–20%) in 31.6%, and high (>20%) in 7.6%. Multivariate analysis showed that the mean 10-year hard CHD risk was 8.76±6.92 (10.82±6.83 in men and 3.18±2.90 in women). Ten-year hard CHD risk was higher in patients with the metabolic syndrome (odds ratio [OR] 2.67, confidence interval [CI] 1.54–4.64), a longer duration of schizophrenia (OR 1.03, CI 1.01–1.05), a history of other medical illnesses (OR 2.02, CI 1.18–3.47), and in those participating in art therapy (OR 2.13, CI 1.25–3.64) or therapeutic education (OR 1.93, CI 0.93–4.01). Ten-year hard CHD risk was lower in patients receiving risperidone (OR 0.23, CI 0.08–0.68), any anti-epileptic (OR 0.41, CI 0.24–0.73), or any benzodiazepine (OR 0.33, CI 0.17–0.66) medication.

**Conclusion:**

CHD is prevalent in patients with schizophrenia in Lebanon. Physicians are recommended to monitor the components of the metabolic syndrome to identify patients with increased risk of cardiovascular diseases.

## Introduction

Life expectancy of patients with schizophrenia remains lower than that of the general population [1–3]. This could be attributed to the increased cardiovascular mortality rates in these patients, rather than suicidal risk alone [4–8]. Several factors can explain the increased mortality risk such as the effect of antipsychotic medications and the high prevalence of the traditional coronary heart disease (CHD) risk factors, mainly obesity and an unhealthy lifestyle [1,6–8]. In fact, patients with schizophrenia have been shown to have sedentary behaviors [9–11], lower activity and exercise levels, and unhealthy dietary habits compared with the general population. It is well known that a low level of physical activity and a high calorie diet reduce life expectancy and increase the risk of both the metabolic syndrome and CHD in the general population, as well as in patients with schizophrenia [9–12]. Furthermore, smoking is much more prevalent in patients with schizophrenia than in the general population or in patients with other mental disorders [13,14]. Treatment with antipsychotic medications in patients with schizophrenia is essential for the improvement of their psychiatric symptoms and prognosis. However, one of the established side effects of antipsychotics (predominantly the atypical ones) is weight gain, which is a major risk factor for the metabolic syndrome and cardiovascular disease (CVD) [5,8,15–17].

To estimate the risk of developing CVD, it is essential to use a cardiovascular risk stratification tool. The Framingham risk score (FRS) provides a validated calculation of the 10-year risk of developing coronary vascular disease [18]. Few studies have assessed the risk of future CHD in patients with schizophrenia using a risk estimator [19–22]. Correll *et al.* reported a 10-year CHD risk of 6.5% for inpatients with schizophrenia (*n*=111). They also reported that 23.4% of patients with schizophrenia had a CHD risk ≥10% [23]. Goff *et al.* [20] showed that the 10-year risk of CHD was significantly greater in patients with schizophrenia than in the general population (9.4% vs. 6.3% in males and 7.0% vs. 4.2% in females). Others have reported similar findings, with 10-year CHD risks of between 6.5% and 7.2% in patients with schizophrenia [19–24].

In Lebanon, the prevalence of CHD in the general population is 47%, as reported by the World Health Organization (WHO) in 2014 [25]; however, the prevalence and predictors of cardiovascular risk among the general population and, especially in patients with schizophrenia receiving antipsychotics drugs, remain unknown. Therefore, we conducted the current study to evaluate the cardiovascular risk profile of Lebanese patients with schizophrenia and to determine the modifiable and non-modifiable factors affecting this risk.

## Materials and methods

### Study design

A cross-sectional study was conducted between April and July 2016 at the Psychiatric Hospital of the Cross, the largest center for the management of psychiatric disorders in Lebanon. The study protocol was reviewed and approved by the research and ethics committee of the hospital. Participants or their legal representative provided written informed consent before entering the study.

Inclusion criteria were long-term hospitalized inpatients aged 20–75 years, without any CHD at baseline examination, with a diagnosis of schizophrenia according to the Diagnostic and Statistical Manual of Mental Disorder “Fifth Edition” (DSM-V) [26], and receiving at least one antipsychotic agent for longer than 6 weeks. Exclusion criteria were patients treated with a statin and those with thyroid disorders. Statins are a leading therapeutic class of lipid-lowering agents and are established in the primary and secondary prevention of coronary artery disease [27]; lower lipid levels in patients treated with statins will lower the estimated risk of hard CHD [27]; CHD can be classified as either “hard” (e.g. myocardial infarction [MI], CHD death) or “soft” (e.g. chest pain) [28]. Hyper- and hypothyroidism can produce changes in cardiac contractility, myocardial oxygen consumption, cardiac output, blood pressure, and systemic vascular resistance, thus predisposing a patient to CHD [29]. Furthermore, thyroid disorder can affect lipid levels and thus change the risk estimation of hard CHD [29].

### Sample size calculation

Sample size calculation was performed using Epi Info software (version 7.07 Centers for Disease Control and Prevention, Atlanta, GA, USA) [30]. Based on: (i) a population size of 40,000 psychiatric patients in Lebanon; (ii) an expected risk of 10-year hard CHD ≥10% of 23.4% in patients with schizophrenia [23]; and (iii) 5% confidence limits [30], we calculated a sample size of 274 patients to allow for adequate power (80%) for bivariate analysis. However, we included 400 patients to account for refusals.

### Data collection

#### Medical files

Medical files were searched for the following information: (i) demographics, including age, sex, geographic region, marital status, occupation, education level, and total monthly salary per household (divided into three levels: low, USD <1,000; intermediate, USD 1,000–2,000; and high, USD >2,000); (ii) clinical information of the participants, including diagnosis, duration of schizophrenia, and duration of hospitalization; (iii) history of other medical illnesses, including hypertension, asthma, chronic obstructive pulmonary disease, diabetes, epilepsy, and a family history of mental disorders; (iv) medication intake at the time of the study; and (v) social habits of the participants, including smoking status, alcohol intake, and diet. Diet was recorded as a dichotomous variable: those who were receiving a special diet (such as low carbohydrate and low fat), and those receiving a regular diet.

#### Questionnaires

Data were also collected using the physical activity questions from the Framingham Study – an originally designed self-reporting questionnaire assessing participants’ daily activities [31]. Participants were asked about their average hours of sleep, rest, occupational, and extracurricular activities (ergotherapy, art therapy, physical activity/sports, therapeutic education, and drawing) over a typical 24-hr period. These are the only extracurricular activities that are allowed for patients at the Psychiatric Hospital of the Cross. Information regarding the intensity of the physical activity was also collected. To test for the effect of each activity on cardiovascular risk, we categorized each activity into a dichotomous variable (yes/no), with a ‘yes’ answer meaning a daily activity of 30 min or more. The questionnaire was administered in Arabic, the native language in Lebanon. The physical activity questionnaire was first translated into Arabic by a translator and then translated back into English by another translator to ensure consistency. The Arabic version of the questionnaire was tested on a pilot sample of 20 patients before the official data collection commenced. Any discrepancies in translation were resolved by the study investigators, in agreement with the translators. Of note, the pilot sample of 20 patients was included in the final database and analysis.

### Metabolic syndrome

The National Cholesterol Education Program Adult Treatment Panel III (NCEP/ATP III) definition was used for the metabolic syndrome [32]. The metabolic syndrome is defined as the presence of three or more of the following risk factors: (i) waist circumference >102 cm (40 in) in men or >88 cm (35 in) in women; (ii) blood pressure ≥130/85 mmHg; (iii) fasting triglyceride level ≥150 mg/dL; (iv) fasting high-density lipoprotein (HDL) cholesterol level <40 mg/dL (in men) or <50 mg/dL (in women); and (v) fasting blood glucose level ≥110 mg/dL [32]. Patients were categorized into two groups: those with two or fewer risk factors (not fulfilling the criteria of having metabolic syndrome) and those with three or more risk factors (fulfilling the criteria of having metabolic syndrome).

### Ten-year CHD risk calculation

The Framingham/ATP III criteria were used to estimate the 10-year risk of hard CHD [33]. The NCEP/ATP III risk prediction algorithm was proposed for people aged 20–75 years, and thus, only participants in this age range were included in the present analyses. Calculation of the Framingham 10-year risk of hard CHD included the following factors: age, total cholesterol, HDL cholesterol, systolic blood pressure, treatment for hypertension, and smoking status. Patients were assigned to one of three categories according to their 10-year hard CHD risk: <10% (low), 10–20% (intermediate), and >20% (high). Type 2 diabetes was considered a CHD risk equivalent. Thus, patients with type 2 diabetes were considered to have a hard CHD risk equivalent to that of patients with existing CHD (i.e. 10-year risk >20%) [33,34].

### Laboratory analysis

Blood samples were drawn from the antecubital vein between 06:00 and 07:00 hr after an overnight fast (12 hr of fasting) to measure lipid and glucose levels. Total cholesterol, HDL cholesterol, low-density lipoprotein cholesterol, triglycerides, and fasting blood glucose were measured by COBAS INTEGRA^®^ 400 plus analyzer (Roche Diagnostics GmbH, Mannheim, Germany).

### Anthropometric measures

Anthropometric measures, including height, weight and waist circumference, were made on the same day as the blood collection, using the Detecto 339 physician scale and a tape measure. These measures were taken by hospital nurses who received appropriate training prior to the beginning of data collection.

Body mass index (BMI) was calculated as body weight in kilograms divided by the square of the height in meters (kg/m^2^), according to the European Society of Cardiology (ESC)/European Atherosclerosis Society (EAS) guidelines 2011 [35] and WHO [36]. BMI was classified into four categories: underweight (<18.5 kg/m^2^), ­normal (18.5–24.9 kg/m^2^), overweight (25.0–29.9 kg/m^2^), and obese (≥30.0 kg/m^2^) [37].

Waist circumference was considered normal if <102 cm for men and <88 cm for women, according to the NCEP/ATP III guidelines for the definition of the metabolic syndrome [32].

Blood pressure was measured by qualified registered nurses using a digital sphygmomanometer (ALP-K2, Zhejiang, China). Two blood pressure measurements were taken in a seated position at the upper arm, at 1–2 min intervals, according to NCEP [32] and JNC7 (Seventh Joint National Committee) guidelines [37]. The average of the two readings was used to represent the patient’s blood pressure. Blood pressure was considered uncontrolled/high when systolic blood pressure was ≥140 mmHg or diastolic blood pressure was ≥90 mmHg [37]. Patients were also considered hypertensive if they were treated with an antihypertensive medication according to their medical file. However, we did not document the type of antihypertensive medications taken.

### Data analysis

Data entry and analysis were performed using the ­Statistical Package for the Social Sciences (SPSS) software version 22. The independent-sample *t*-test was used to compare means between two groups (hard CHD <10% and ≥10%). Pearson correlation coefficient was used to correlate between quantitative variables. For categorical variables, the χ^2^ and Fisher exact tests were used where applicable. Multivariate logistic regression and linear regression analyses were conducted using variables with *p*<0.2 in the bivariate analysis [38,39]; potential confounders were eliminated only if *p*>0.2, in order to protect against residual confounding [40]. In the logistic regression analysis, the dichotomous hard CHD (<10% and ≥10%) was used as the dependent variable. A linear regression was also conducted with the 10-year hard CHD risk as a continuous dependent variable. Significance was defined as *p*<0.05.

## Results

### Baseline characteristics of the study sample

Of the 400 patients invited, 329 (82.3%) agreed to participate. The sociodemographic and clinical characteristics of the study population are shown in Table 1. The population had a mean age of 52 years (range 31–74 years) and was primarily from Mount Lebanon (32.2%) and Beirut (19.5%). The majority of patients were male (72.9%), unemployed (74.8%), had a low socioeconomic status (79.0%), were single (85.7%), and had an intermediate level of education (65.7%). Just over half (51.1%) of the patients had a normal BMI, while 48.4% were overweight or obese. Most (64.8%) patients had two or fewer risk factors for the metabolic syndrome, while 35.2% had three or more. Many of the patients also had comorbidity: 42.0% had hypertension, 9.6% had asthma, 11.3% had chronic obstructive pulmonary disease and 15.0% had diabetes. The mean duration of hospital stay was 11.5 years and the mean duration of schizophrenia was 25 years.

The 10-year risk of hard CHD was low (<10%) in 60.8% of the patients, intermediate (10–20%) in 31.6%, and high (>20%) in 7.6%. Overall, the mean 10-year risk of hard CHD was 8.8±6.9: 10.82±6.83 in men and 3.2±2.9 in women. The majority (91.2%) of patients were taking typical antipsychotics, 31.6% were taking atypical antipsychotics, and 24.0% were taking a combination of both (Table 1). Other medications included anti-epileptics, antidepressants, anticholinergics, benzodiazepines, and sedating antihistamines. Anti-epileptics were commonly used as mood stabilizers in patients with schizoaffective disorder. Antidepressants were used in treating the depressive symptoms of schizoaffective disorder. Benzodiazepines and antihistamines were used for their anxiolytic effect, and anticholinergics for the extrapyramidal and dyskinetic manifestations associated with neuroleptic use.

### Bivariate analysis

Variables that were associated with low (<10%) and high (≥10%) hard CHD risk are shown in Table 2. Female sex, family history of mental disorders, and the use of risperidone, clozapine, anti-epileptics, and benzodiazepines, were associated with a significantly lower risk of hard CHD. In contrast, older age, a longer duration of schizophrenia, smoking, participating in art therapy, having a special diet, and fulfilling the criteria of the metabolic syndrome were significantly associated with a high risk of hard CHD. No significant differences were found between both groups for ergotherapy, therapeutic education, sports, drawing activity, and employment variables. All other variables examined were not associated with hard CHD risk (*p*>0.2).

### Multivariate analysis

A backward logistic regression analysis using the two-category hard CHD showed that several variables were associated with a significantly greater risk of high (≥10%) hard CHD, including the metabolic syndrome, having a history of other medical illnesses, and participating in art therapy or therapeutic education (Table 3). Furthermore, high CHD risk was increased significantly by 3.3% for each year since diagnosis of schizophrenia illness (Table 3). In contrast, high CHD risk was significantly lower in patients receiving risperidone, any anti-epileptic, or any benzodiazepine (Table 3).

Results from the linear regression analysis were similar to those observed in the logistic regression analysis and are shown in Supplementary Table 1.

## Discussion

This is the first study conducted in Lebanon to investigate predictors and risk of CVD in patients with schizophrenia receiving antipsychotic medication. Our results showed that Lebanese patients with schizophrenia, particularly males, have an increased CHD risk, similar to findings reported in the USA [20,22]. Cohn *et al*. also reported an increased 10-year cardiac risk by 30% in men with schizophrenia [22]. Additionally, Bobes *et al*. showed that the overall risk of CHD in 10 years was 6.8% in patients with schizophrenia; a risk that was also significantly greater in males than in females (8.3% vs. 4.5%, *p*<0.001) [21]. Goff *et al*. showed that the 10-year risk of CHD was significantly higher in both male (9.4% vs. 7.0%) and female (6.3% vs. 4.2%) schizophrenia patients compared with controls [20]. Findings from the CATIE (Clinical Antipsychotic Trials of Intervention Effectiveness) schizophrenia study showed that the overall change from baseline in 10-year CHD risk was significant in men (*p*=0.017) but not in women [41]. The disparity in CHD risk between sexes might be related to the trend in men towards a higher BMI – and subsequently higher risk of hypertension and diabetes, and thus, CHD risk – that has been observed in males compared with females with schizophrenia [42].

The etiology of this increased CHD risk is multifactorial, and includes both modifiable and non-modifiable risk factors. These factors, except for the metabolic syndrome, are particularly important given our knowledge about the factors predictive of 10-year CHD risk in patients with schizophrenia is scarce. 

### Non-modifiable risk factors

Our results showed that non-modifiable risk factors, including a longer duration of schizophrenia illness and a history of other medical illnesses, increase the 10-year risk of hard CHD. Similarly, it has previously been reported that psychiatric patients with a long duration of illness have an increased risk of CHD [20], probably because of the use of antipsychotic medications that increase risk factors for CHD, as explained in more detail below [15,19,21,43]. Furthermore, people with severe mental illness, such as schizophrenia, bipolar, schizoaffective disorders, or major depressive disorders, are significantly more likely to have several modifiable risk factors for CVD compared with the general population (for review see [44]). Patients with a severe mental illness are also more likely to be overweight, smoke, and have diabetes, hypertension, dyslipidemia, and poor physical illness [15,20,45].

### Modifiable risk factors

The presence of the metabolic syndrome in patients with schizophrenia was associated with a 10-year risk of hard CHD >20%. This finding is in accordance with previous studies assessing the relationship between the prevalence of the metabolic syndrome and risk of CHD in hospitalized and non-hospitalized patients treated with antipsychotic medications [15,19,21,43]. Calculation of hard CHD risk and the metabolic syndrome include modifiable factors that increase cardiac disease risk in patients with schizophrenia [20,46]. In our study, these modifiable factors were significantly associated with an elevated risk of 10-year hard CHD.

There is considerable evidence indicating that certain antipsychotic medications, particularly those in the atypical (second generation) class, increase the risk of CHD in patients with schizophrenia [20,41,47], most likely through an association with weight gain and adverse alterations in glucose and lipid metabolism [45]. However, differences may exist among the individual medications [45]. Risperidone, for example, has little or no adverse effects on triglyceride and total cholesterol levels [45,48–52], but has an intermediate risk of weight gain and glucose elevation when compared with other atypical, as well as typical, antipsychotic medications [45,52]. In contrast, clozapine appears to be associated with a high risk of weight gain, type 2 diabetes mellitus, and dyslipidemia [22,45]. In our study, both risperidone and clozapine were associated with a low 10-year CHD risk, despite the known differences in CVD risk profiles between the two medications. To date, very few studies have specifically examined an association between antipsychotic medications and 10-year CHD risk [22,41,47,53]. In accordance with our findings, the CATIE trial demonstrated a decreased 10-year CHD risk with risperidone [41]. Cohn *et al*. [22], who evaluated the Framingham 10-year risk of MI, did not observe a significant difference in risk between patients taking risperidone and clozapine. Similarly, CVD mortality risk did not differ significantly between risperidone and clozapine in the study by Kelly *et al.* [47]. Enger *et al*. [53] showed that the use of both typical and atypical antipsychotic medications reduced the risk of MI, despite both classes equally increasing the risk of new-onset diabetes. Increased CHD risk in patients with schizophrenia is complex, and likely to involve more than the effect of antipsychotic medications alone.

The use of older-generation anti-epileptic medications, which are highly hepatic enzyme inducing through the activation of the cytochrome P450 system – which is involved in the synthesis of serum cholesterol – are known to have adverse effects on multiple surrogate markers of vascular risk, such as lipid metabolism [54–56], and may therefore increase the risk of vascular events and mortality [57–59]. However, in our study, the 10-year risk of hard CHD in patients receiving anti-epileptic medications was low, possibly due to some epileptic medications that inhibit metabolizing enzymes and might have an effect of decreasing the lipid metabolism, thereby decreasing the production of cholesterol [55]. To our knowledge, no study has specifically investigated the effect of anti-epileptic medications on CHD risk in patients with schizophrenia. However, several epidemiologic studies have shown positive correlations between epilepsy and comorbid vascular disease [60–62].

We also reported a low risk of 10-year hard CHD in patients who received benzodiazepines, which are known to inhibit the sympathetic nervous system, thereby reducing stress, lowering blood pressure, and, possibly leading to a protective effect against CVD risk [63]. Further studies are needed to specifically assess a relationship between benzodiazepines and CHD risk.

The majority of the patients in our study were unemployed, and some practiced therapeutic education and art therapy. It is known that patients with schizophrenia are less likely to engage in physical activity and are more likely to have sedentary behaviors compared with the general population [64]. We demonstrated that therapeutic education and art therapy were associated with a high (≥10%) 10-year hard CHD risk. These findings confirm other studies in which a sedentary lifestyle in patients with schizophrenia has been linked to CHD risk [65,66]. Many patients might also lack sufficient motivation to increase their levels of physical activity [64]. However, even when adequate facilities for physical activities are available to patients, they do not necessarily take advantage of them [12].

### Limitations

Our study has several limitations. Firstly, the study was conducted at a single center and was confined to inpatients with chronic disease. It may not, therefore, reflect the true cardiovascular risk of all patients with schizophrenia. Secondly, the cross-sectional design does not permit the evaluation of the effect of concomitant treatments and its correlation with cardiac risk factors, and does not permit comparison with other populations, such as patients without a mental health disorder or the general population. Randomized, prospective trials are necessary to specifically examine possible differential effects of particular antipsychotics on cardiac risk factors. The relationship between dose and duration of medications and 10-year risk of developing CHD was not studied. Finally, lifestyle activity was evaluated through a direct interview; thus, the information collected directly from patients could have been affected by the low level of cognition and the negative effects of antipsychotic treatment.

## Conclusion

Patients with schizophrenia have a high risk of developing CHD and should be closely monitored to prevent, reduce, and manage individual risk factors, particularly those that are modifiable, such as lifestyle factors. Furthermore, there is a need for active collaboration between the treating psychiatrist and the cardiologist to address the various CHD risk factors to reduce the risks. Increased awareness among mental health professionals about the various cardiometabolic risk factors in patients with schizophrenia is also recommended.

## Figures and Tables

**Table 1:** Sociodemographic characteristics of the sample population.

Characteristic	Mean±SD or *n* (%)
Age, years	52.0±12.8
Duration of schizophrenia, years	25.0±13.1
Duration of hospitalization, years	11.5±11.2
Sex	
Male	240 (72.9)
Female	89 (27.1)
Region	
Mont Lebanon	106 (32.2)
Beirut	64 (19.5)
South	43 (13.0)
North	45 (13.7)
Beqaa	45 (13.7)
Foreign	26 (7.9)
Marital status	
Single	282 (85.7)
Married	28 (8.5)
Divorced	19 (5.8)
Employment	
Unemployed	246 (74.8)
Employed	83 (25.2)
Education level^a^	
Illiterate	22 (6.7)
Primary	114 (34.7)
Complementary	102 (31.0)
Secondary	59 (17.9)
University	32 (9.7)
Monthly salary^b^	
Low	260 (79.0)
Intermediate	66 (20.1)
High	3 (0.9)
Body mass index^c^	
Underweight	2 (0.5)
Normal	168 (51.1)
Overweight	94 (28.6)
Obese	65 (19.8)
Metabolic syndrome^d^	
1 risk factor	73 (22.2)
Any 2 risk factors	140 (42.6)
Any 3 risk factors	94 (28.6)
Any 4 risk factors	22 (6.6)
5 risk factors	0 (0.0)
Presence of other medical illnesses	
Hypertension	138 (42.0)
Asthma	31 (9.56)
Chronic obstructive pulmonary disease	37 (11.3)
Diabetes	49 (15.0)
Medications	
Typical antipsychotics	300 (91.2)
Atypical antipsychotics	104 (31.6)
Both typical and atypical	79 (24.0)

^a^Illiterate: unable to read and write; primary education: <8 years; complementary: 8–15 years; secondary: beginning at age 15 years until university level; university: of sufficient level to start higher education.^b^ Low, USD <1,000 USD; intermediate, USD 1,000–2,000; and high USD >2,000.^c^Underweight, <18.5 kg/m^2^; normal, 18.5–24.9 kg/m^2^; overweight, 25.0–29.9 kg/m^2^; and obese, ≥30.0 kg/m^2^.^d^Having three or more of the following risk factors: (i) waist circumference >102 cm (>40 in) in men or >88 cm (35 in) in women; (ii) blood pressure ≥130/85 mmHg; (iii) fasting triglyceride ≥150 mg/dL; (iv) fasting high-density lipoprotein cholesterol level <40 mg/dL (in men) or <50 mg/dL (in women); and (v) fasting blood glucose ≥110 mg/dL.SD, standard deviation; USD, United States dollars.

**Table 2:** Bivariate analysis of 10-year risk of hard coronary heart disease (CHD).

Variable	10-year hard CHD risk, mean±SD or n (%)		*p*	OR	95% CI
	Low risk (<10%), *n*=200 (60.8%)	High risk (≥10%), *n*=129 (39.2%)			
Age, years	47.32±12.20	59.08±10.07	<0.001	1.093	1.068–1.119
Duration of illness, years	22.55±12.75	28.82±12.75	<0.001	1.039	1.020–1.057
Duration of hospitalization, years	10.14±10.74	13.71±11.52	0.004	1.029	1.009–1.050
Sex					
Male	114 (47.5)	126 (52.5)	<0.001	0.032	0.010–0.103
Female	86 (96.6)	3 (3.4)			
Family history of mental disorders					
No	125 (56.6)	96 (43.4)	0.025	0.573	0.352–0.934
Yes	75 (69.4)	33 (30.6)			
History of other medical illnesses					
No	142 (66.0)	73 (34.0)	0.007	1.878	1.182–2.984
Yes	58 (50.9)	56 (49.1)			
Clozapine					
No	157 (57.7)	115 (42.3)	0.013	0.444	0.232–0.851
Yes	43 (75.4)	14 (24.6)			
Risperidone					
No	174 (58.4)	124 (41.6)	0.006	0.270	0.101–0.722
Yes	26 (83.9)	5 (16.1)			
Anti-epileptic medications					
No	101 (51.3)	96 (48.7)	<0.001	0.351	0.216–0.568
Yes	99 (75.0)	33 (25.0)			
Benzodiazepine					
No	137 (54.6)	114 (45.4)	<0.001	0.286	0.155–0.529
Yes	63 (80.8)	15 (19.2)			
Smoking					
No	27 (77.1)	8 (22.9)	0.036	2.361	1.037–5.373
Yes	173 (58.8)	121 (41.2)			
Ergotherapy					
No	174 (60.0)	116 (40.0)	0.095	0.750	0.370–1.519
Yes	26 (66.7)	13 (33.3)			
Therapeutic education					
No	177 (62.8)	105 (37.2)	0.072	1.759	0.946–3.272
Yes	23 (48.9)	24 (51.1)			
Art therapy					
No	137 (65.2)	73 (34.8)	0.028	1.668	1.055–2.639
Yes	63 (52.9)	56 (47.1)			
Physical activity/sports					
No	102 (51.0)	65 (50.4)	0.914	1.025	0.658–1.596
Yes	98 (49.0)	64 (49.6)			
Drawing activity					
No	194 (97.0)	123 (95.3)	0.435	1.577	0.497–5.001
Yes	6 (3.0)	6 (4.7)			
Dietary pattern					
Regular diet	185 (63.1)	108 (36.9)	0.013	2.398	1.186–4.848
Special diet	15 (41.7)	21 (58.3)			
Employment					
Unemployed	155 (63.0)	91 (37.0)	0.156	1.438	0.869–2.380
Employed	45 (54.2)	38 (45.8)			
Metabolic syndrome					
No	141 (66.2)	72 (33.8)	0.006	1.892	1.192–3.002
Yes	59 (50.9)	57 (49.1)			

CI, confidence interval; OR, odds ratio; SD, standard deviation.

**Table 3:** Multivariate logistic regression analysis of variables associated with high (≥10%) 10-year risk of hard coronary heart disease (CHD).^a^

Variable	OR	95% CI	*p*
History of other medical illnesses	2.022	1.180, 3.465	0.010
Risperidone	0.226	0.075, 0.682	0.008
Anti-epileptic medications	0.415	0.236, 0.729	0.002
Benzodiazepine	0.333	0.169, 0.658	0.002
Therapeutic education	1.931	0.930, 4.008	0.077
Art therapy	2.129	1.247, 3.637	0.006
Metabolic syndrome^b^	2.672	1.540, 4.637	<0.001
Duration of schizophrenia illness	1.033	1.011, 1.054	0.002

^a^The dichotomous hard CHD (<10% and ≥10%) was used as the dependent variable. ^b^Two or fewer risk factors vs. three or more risk factors. Variables entered in the logistic regression included sex, family history of mental disorders, history of other medical illnesses, risperidone, clozapine, anti-epileptic medication, benzodiazepine, smoking, ergotherapy, therapeutic education, art therapy, dietary intake, employment, metabolic syndrome, duration of illness, duration of hospitalization, and age.CI, confidence interval; OR, odds ratio.
